# Oxidative Stress Response’s Kinetics after 60 Minutes at Different (30% or 100%) Normobaric Hyperoxia Exposures

**DOI:** 10.3390/ijms24010664

**Published:** 2022-12-30

**Authors:** Clément Leveque, Simona Mrakic-Sposta, Pierre Lafère, Alessandra Vezzoli, Peter Germonpré, Alexandre Beer, Stéphane Mievis, Fabio Virgili, Kate Lambrechts, Sigrid Theunissen, François Guerrero, Costantino Balestra

**Affiliations:** 1 Environmental, Occupational, Aging (Integrative) Physiology Laboratory, Haute Ecole Bruxelles-Brabant (HE2B), 1160 Brussels, Belgium; 2 Laboratoire ORPHY, EA 4324, Université de Bretagne Occidentale, 29238 Brest, France; 3 Institute of Clinical Physiology, National Research Council (CNR), 20162 Milan, Italy; 4 DAN Europe Research Division (Roseto-Brussels), 1020 Brussels, Belgium; 5 Hyperbaric Centre, Queen Astrid Military Hospital, 1120 Brussels, Belgium; 6 Council for Agricultural Research and Economics-Food and Nutrition Research Centre (C.R.E.A.-AN), Rome, Italy; 7 Anatomical Research and Clinical Studies, Vrije Universiteit Brussels (VUB), 1090 Brussels, Belgium; 8 Motor Sciences Department, Physical Activity Teaching Unit, Université Libre de Bruxelles (ULB), 1050 Brussels, Belgium

**Keywords:** normobaric oxygen paradox, hyperoxic–hypoxic paradox, hyperoxia, oxygen biology, cellular reactions, human, oxygen therapy, human performance, decompression, diving

## Abstract

Oxygen is a powerful trigger for cellular reactions and is used in many pathologies, including oxidative stress. However, the effects of oxygen over time and at different partial pressures remain poorly understood. In this study, the metabolic responses of normobaric oxygen intake for 1 h to mild (30%) and high (100%) inspired fractions were investigated. Fourteen healthy non-smoking subjects (7 males and 7 females; age: 29.9 ± 11.1 years, height: 168.2 ± 9.37 cm; weight: 64.4 ± 12.3 kg; BMI: 22.7 ± 4.1) were randomly assigned in the two groups. Blood samples were taken before the intake at 30 min, 2 h, 8 h, 24 h, and 48 h after the single oxygen exposure. The level of oxidation was evaluated by the rate of reactive oxygen species (ROS) and the levels of isoprostane. Antioxidant reactions were observed by total antioxidant capacity (TAC), superoxide dismutase (SOD), and catalase (CAT). The inflammatory response was measured using interleukin-6 (IL-6), neopterin, creatinine, and urates. Oxidation markers increased from 30 min on to reach a peak at 8 h. From 8 h post intake, the markers of inflammation took over, and more significantly with 100% than with 30%. This study suggests a biphasic response over time characterized by an initial “permissive oxidation” followed by increased inflammation. The antioxidant protection system seems not to be the leading actor in the first place. The kinetics of enzymatic reactions need to be better studied to establish therapeutic, training, or rehabilitation protocols aiming at a more targeted use of oxygen.

## 1. Introduction

Oxygen, the third most abundant element in the biosphere, is mostly known for its role in the development and ability to sustain aerobic organisms [1], and it is administered as a therapeutic agent [2]. Indeed, oxygen is considered as a cornerstone of modern medical care within well-defined limits to mitigate adverse effects [3]. Nowadays, oxygen use is not only limited to counteract hypoxia or restore patient saturation. New research paths have coined the use of several inhaled oxygen fractions (FiO_2_) in the physiology of aging [4,5], such as cartilage degeneration [6], prediabetes [7], or neuroprotection [8]. It must be acknowledged that variable FiO_2_, hypoxic or hyperoxic, is already relatively common, for instance, for preconditioning in cardiovascular disease [9,10], sports training [11], and before exposure to challenging environments such as scuba diving [12], high altitude military parachute freefall [13,14], and space flight activities [15].

Under physiological conditions, unstable molecules derived from oxygen called reactive oxygen species (ROS; i.e., superoxide anions (O2•–), hydroxyl radicals (•OH), peroxyl radicals (ROO•), and alkoxy radicals (RO•)), are primary signals to modulate several pathways involved in homeostasis and cell proliferation/differentiation/survival [16,17]. 

ROS increase due to environmental and/or chemical stimuli. When ROS increase and the endogenous antioxidant system (enzymatic and/or non-enzymatic) is perturbed, oxidative stress is triggered. This results in damage to lipids, proteins, and DNA [18,19,20].

Furthermore, it is known that oxidative stress, inflammation, the immune system, and metabolism are intricately intertwined [21,22]. Specific inflammatory cytokines utilize ROS as part of their signaling cascades, and these can contribute to the development and/or progression of acute or chronic inflammatory responses [23].

Our previous works highlighted the non-linearity of the dose–response curve, which makes the definition of an optimal oxygen dose difficult. 

Based on the current literature, several factors such as FiO_2_ [11,15,24,25], duration and frequency [26,27,28], or pressure of exposure—either normobaric, hypobaric, or hyperbaric [15,26,29]—may influence the ability to reach a specific clinical or molecular effect. 

Although some mechanisms were identified, there is a lack of data regarding the kinetics of the cellular response to those different oxygen partial pressures. 

Therefore, the objective of this study is to describe the kinetics of cell expression in response to a single exposure at different FiO_2_ percentages (30% and 100%) in normobaric conditions only.

## 2. Results

### 2.1. Reactive Oxygen Species (ROS) Rate and Isoprostane Levels after One Hour of Oxygen Exposure at a FiO_2_ of 30% or 100%

**Figure 1:**
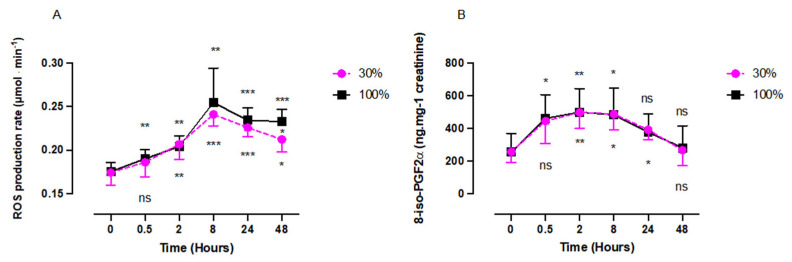
Evolution of ROS (**A**) production rate and isoprostane level (**B**) after 60 min of mild (30%, n = 6) or high (100%, n = 8) hyperoxia. Results are expressed as mean ± SD. T0 represents pre-exposure baseline (ns: not significant; *: *p* < 0.05, **: *p* < 0.01, ***: *p* < 0.001; intragroup: one-way ANOVA; intergroup: unpaired *t* test).

Both oxygen exposures, 30% and 100%, elicited a significant increase of plasmatic ROS production rate (Figure 1A). Both responses were similar in amplitude and shape, characterized by a significant increase with a peak at 8 h after exposure (30%: 0.24 ± 0.01 µmol.min^–1^; 100%: 0.25 ± 0.04 µmol.min^–1^) followed by a decrease after 30%, reaching a plateau after 100%. At 48 h, there was a significant difference between oxygen concentrations (30%: 0.21 ± 0.01 µmol.min^–1^; 100%: 0.23 ± 0.01 µmol.min^–1^; *p* = 0.012, unpaired *t* test). Isoprostane (8-iso-PGF 2α), a biomarker of lipid peroxidation (Figure 1B), followed the same trend for both oxygen exposures with a peak at 2 h after 30% exposure (498.7 ± 99.55 pg.mg^–1^; 100%: 499.1 ± 164.0 pg.mg^–1^; *p* = 0.0078). At 48 h the baseline was reached for both exposures (30%: 266.4 ± 95.67 pg.mg^–1^; 100%: 281.2 ± 135.3 pg.mg^–1^; *p* = 0.63 and 0.99, respectively).

### 2.2. Antioxidant Response (TAC, SOD, CAT) after One Hour of Oxygen Exposure at a FiO_2_ of 30% or 100%

**Figure 2:**
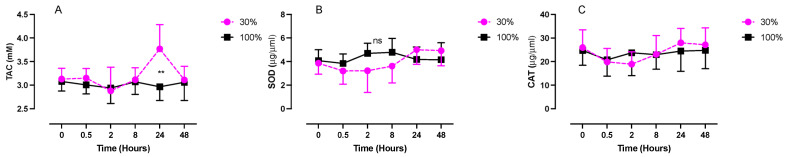
Evolution of the antioxidant response after 60 min of mild (30%, n = 6) or high hyperoxia (100%, n = 8). (**A**) Total antioxidant capacity (TAC). (**B**) Superoxide dismutase activity (SOD). (**C**) Catalase activity (CAT). Results are expressed as mean ± SD. T0 represents pre-exposure (ns: not significant, **: *p* < 0.01; intragroup one-way ANOVA; intergroup unpaired *t* test).

Figure 2 illustrates the antioxidant response as a function of blood total antioxidant capacity (TAC), superoxide dismutase (SOD), and catalase (CAT) concentration. SOD and CAT activities were not modified by oxygen exposure (30%: 3.9 ± 0.9 U/mL and 25.9 ± 7.5 U/mL, respectively; 100%: 4.1 ± 0.9 U/mL and 24.7 ± 6.3 U/mL, respectively). Nonetheless, compared to 100%, after being exposed at 30%, the TAC was significantly increased 24 h after the end of exposure (3.8 ± 0.5 M vs. 2.9 ± 0.3 mM; *p* = 0.004, unpaired *t* test). However, compared to baseline, this difference was not significant (*p* = 0.32, one-way ANOVA).

### 2.3. Inflammatory Response (IL-6, Neopterin, Creatinine, and Urates) after One Hour of Oxygen Exposure at a FiO_2_ of 30% or 100%

**Figure 3:**
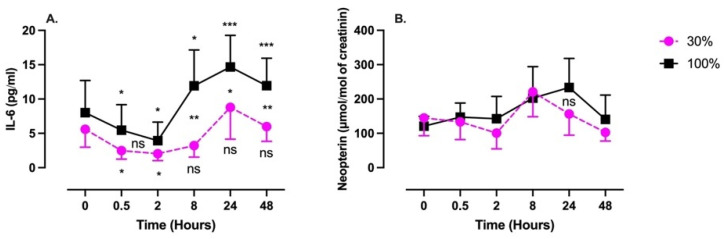
Evolution of the inflammatory response after 60 min of mild (30%, n = 6) or high hyperoxia (100%, n = 8). (**A**) Interleukin-6 (IL-6). (**B**) Neopterin. Results are expressed as mean ± SD. T0 represents pre-exposure (ns: not significant; *: *p* < 0.05, **: *p* < 0.01, ***: *p* < 0.001; intragroup: one-way ANOVA; intergroup: unpaired *t* test).

Interleukin 6 (IL-6) was measured in plasma samples, while neopterin, creatinine, and urates were obtained from urine samples (Figure 3). 

IL-6 followed a similar pattern in both conditions, although with a late higher amplitude after 100% exposure. It was characterized by a significant decrease in concentration followed by a rebound without returning to the baseline. The nadir was reach after 2 h (30%: 2.0 ± 1.0 pg/mL; 100%: 3.9 ± 2.7 pg/mL; *p* = 0.017 and *p* = 0.015, respectively, one-way ANOVA), while the acme was reached after 24 h (30%: 8.8 ± 4.6 pg/mL; 100%: 14.7 ± 2.7 pg/mL; *p* > 0.99 and *p* = 0.001, respectively, one-way ANOVA). 

From 8 h post-exposure and beyond, the two curves significantly diverged (8 h: *p* = 0.001; 24 h: *p* = 0.04; 48 h: *p* = 0.008, unpaired *t* test). 

Neopterin exhibited a similar pattern, although the magnitudes of the changes were reduced. These changes were not statistically significant. 

**Figure 4:**
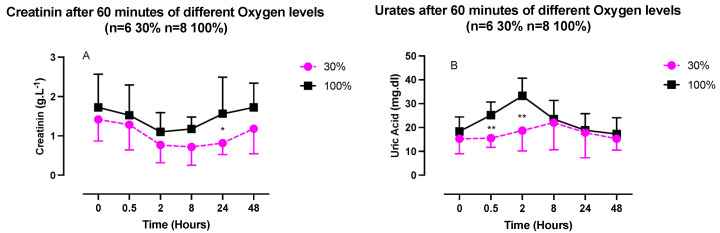
Evolution of urinary markers after 60 min of mild (30%, n = 6) or high hyperoxia (100%, n = 8). (**A**) Creatinine. (**B**) Uric Acid. Results are expressed as mean ± SD. T0 represents pre-exposure (ns: not significant; *: *p* < 0.05, **: *p* < 0.01; intragroup: one-way ANOVA; intergroup: unpaired *t* test).

Creatinine and urates in urine exhibited opposite U-shaped responses, with a creatinine nadir between 2- and 8-h post-exposure and an urate acme within the same time frame (Figure 4), although compared to the baseline, these variations were not statistically significant (one-way ANOVA). There was, however, a significant difference in the urate’s kinetics. It was characterized by a stronger initial response after exposure at 100% (30 min: 25.2 ± 5.5 mg/dL vs. 15.5 ± 3.8 mg/dL, *p* = 0.03; and 2 h, 35.2 ± 7.5 mg/dL vs. 18.7 ± 8.5 mg/dL, *p* = 0.005; unpaired *t* test).

### 2.4. Percent of Expressed Proteins and Reactive Oxygen Species (ROS) after One Hour of Oxygen Exposure at a FiO_2_ of 30% or 100%

**Figure 5:**
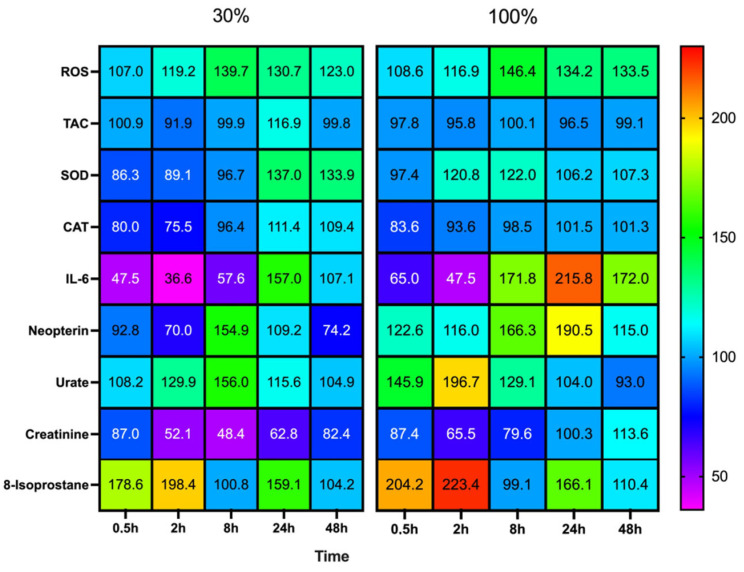
Heat map of percent changes compared to baseline, derived responses after 60 min of mild (30%, n = 6) or high hyperoxia (100%, n = 8). Each measurement is compared to baseline (T0), which was set at 100%. Results are given as mean change.

The magnitude and direction of changes among all results are illustrated in Figure 5.

## 3. Discussion

Hormesis in mammals is defined as an adaptation capacity to different stresses, which encompasses dynamic processes of repair, recovery, and toxicity that occur over time [30]. At the most basic level, it is mediated through “dose–response” and “dose–time” combinations to oxidative stress, metabolic changes, and inflammation [31]. This is a well-known process in pathological situations [32] or ageing. Indeed, the age-related imbalance between pro- and anti-inflammatory factors is a recognized etiology for metabolic, cardiovascular, and neurodegenerative diseases [33]. 

Until a decade ago, ROS were thought to cause exclusively toxic effects and were associated with various pathologies [34]; with time, this view has changed, considering that the presence of ROS in cells indicates that ROS production was evolutionarily selected in order to achieve certain useful roles [35]. It is now accepted that biological specificity of ROS action is achieved through the amount, duration, and localization of their production, as we underline in this study.

More recent data have encouraged us to investigate a new paradigm, namely, to consider oxygen as a potent stimulus of molecular complex reactions rather than a simple drug [36]. 

Several studies exploring the effect of pulsed hyperoxia demonstrated that it is possible to stimulate the production of hypoxia inducible factor (HIF-1α), which was believed to be expressed only under hypoxic conditions—hence its name.

**Figure 6:**
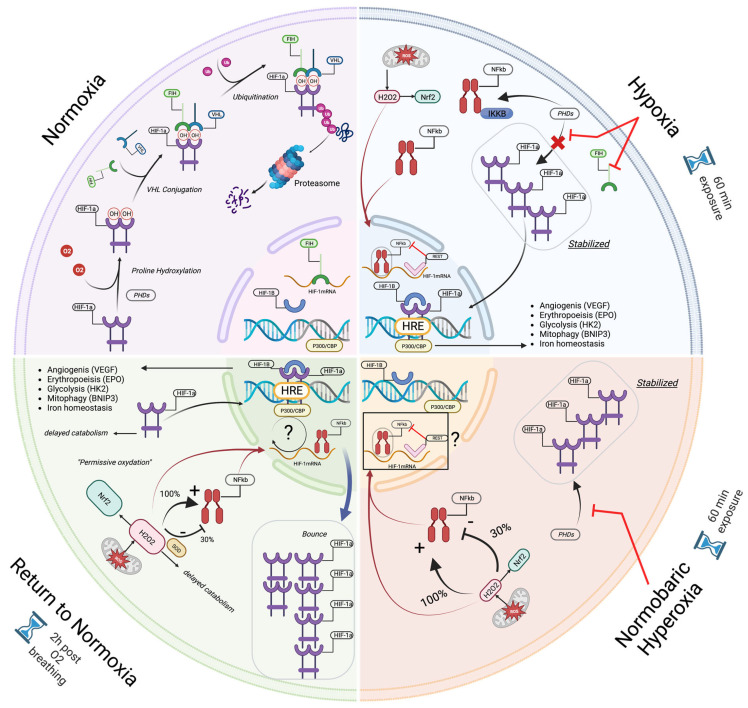
Illustration (created with Biorender.com) of the mechanisms involved during the normobaric oxygen paradox: First, in normoxia, HIF−1α is constantly produced and destroyed by the intervention of PHDs and FIH. During hypoxia and intermittent hyperoxia, the stabilization in the cytosol of HIF−1α protein is improved by repressing the activity of isoforms 1 to 3 of prolyly hydroxylase (PHDs) and of FIH (only in hypoxia), whereas nuclear synthesis is blocked by inhibiting proteins such as REST. Therefore, during these altered oxygen states, immune activation seems to start earlier than antioxidant defenses, allowing subtle kinetics between inflammation and ROS, in their role similar to a “second messenger”. Upon the return to normoxia, the destabilization of HIF−1α by the intervention of PHD and FIH is delayed by the late activation of antioxidant protection, leaving the cell in a state of “permissive oxidation”. Following this, nuclear synthesis begins earlier, explaining the rebound phenomenon encountered in the production of HIF−1α. NRF2 and NF-κB are overexpressed after 100%, whereas under 30% only NRF2 is stimulated.

This phenomenon is called the “normobaric oxygen paradox” [25,26,37] or the “hyperoxic–hypoxic paradox” [24] (for a global view of the mechanisms, see Figure 6).

During these interventions, the return to normoxia after mild hyperoxia is sensed as a hypoxic stress without significant NF-κB activation [29]. On the contrary, high and very high hyperoxia induce a shift toward an oxidative stress response, characterized by NRF2 and NF-κB activation in the first 24 h post-exposure. These observations suggest a modulated cellular response to targeted oxygen exposure [29]. This is further confirmed by microparticle expression under different oxygen partial pressures. All tested exposures demonstrated significant elevations of CD41, CD66b, TMEM, and expression and phalloidin binding, except for 1.4 ATA, which elicited the total opposite response with significant decreases of all measured parameters [15].

As shown in previous studies, oxygen breathing induced a hormetic stimulus, eliciting increased ROS production starting from 30 min post-exposure. 

Our results suggest that there is no difference in the initial response intensity between the two groups (100% and 30%). However, after the acme, which is reached 8 h post-exposure, the kinetics starts to diverge with a between-group statistical difference at 48 h. In addition, the increase of isoprostanes testifies that the production rate of ROS is already significant enough to produce lipid peroxidation. This must be considered together with the kinetics of the antioxidant response of SOD and CAT.

The absence of an intracellular antioxidant response (CAT and SOD), despite the production of ROS over time, suggests that the cell abides a state of “permissive oxidation” and might be explained by the fact that some diurnal variations are possible and also that some circadian rhythms are opposite in males and females for some antioxidants or enzymes [38]; we may consider that since our group was a mix of males and females, at a given point some concomitant situations may result in non-statistically significant changes. 

The significantly higher total antioxidant capacity for a low level of hyperoxia such as 30% recorded at 24 h might be caused by food intake. Furthermore, TAC variations are probably not the most reliable measurement in our experimental setting, given the fact that we followed the participants for 48 h without diet restriction. Some other hyperoxia-induced oxidative stress or transient hypoxia resulting from sleep apnea may also increase myeloid cell recruitment and activation in the lungs. Activated myeloid cells produce myeloperoxidase (MPO), which oxidizes chloride ions to hypochlorous acid, causing oxidative damage and even cell dead [39]. This mechanism, even if important, has been analyzed upon prolonged exposures of several days and may not be preeminent in our experimental setting.

Our results could be explained by the cellular needs for second messengers, such as H_2_O_2_, as mediators likely to act through NRF2 activation, inducing the release of keap1 expression [40]. Although it is now accepted that HIF-1α is the leading agent in the regulation of homeostasis during hypoxia [41] and hyperoxia [26], other regulating factors must be considered, such as NRF2. For instance, repressor element 1-silencing transcription factor (REST) is a known nuclear inhibitor of HIF-1α synthesis [42]. During exposure to hypoxia, REST accumulates in the nucleus with a four-fold increase compared to normoxia. This can be quickly reversed by reoxygenation [43]. 

To the best of our knowledge, the evaluation of REST in hyperoxia has not yet been studied. However, keeping in mind that NOP has the capacity to mimic hypoxia [25,36,44] and is therefore a powerful tool to trigger a hormetic response, it may be used as a reasonable hypothesis to suggests that the lower the hyperoxia delta, the longer the permissive oxidative state, allowing the secondary cellular messengers [45] to potentiate their nuclear transcription role and trigger a strong and rapid response (within the first 3 h). Indeed, recent data demonstrate epigenic changes by methylation/demethylation upon gene expression are related to oxygen homeostasis [46]. The kinetics of these oxygen-induced activation/deactivation mechanisms are dependent upon enzymatic reactions where the role of 2-oxoglutarate dioxygenase seems to be decisive [47].

Alternatively, this could be also explained by a slower kinetics of intracellular defenses. Indeed, we have recently demonstrated that after 30% oxygen exposure, the synthesis of glutathione is effective only after 24 h, whereas after exposure at 100%, its synthesis and release occur earlier, approximately 3 h post-exposure [29].

This latter study also demonstrated that the inflammatory response was different between these two conditions (30% and 100%). Exposure at 30% of oxygen was associated with a significant increase in NRF2 without a significant increase in NF-κB, while an exposure at 100% elicited significant activation of inflammatory pathways mediated by NF-κB [29]. Interestingly, our results are consistent with this observation. First, Il-6 expression within the first two hours post-exposure can be explained by synthesis repression related to parallel NRF2 activation [48]. Second, the greater amplitude of IL-6 expression after the exposure at 100% suggests a shift in the cross-linked pathway of NRF2/NF-κB [49]. Although not significant, neopterin shares a similar pattern from inhibition to overexpression with a return to baseline within 24 h. Since this molecule is indicative of a pro-inflammatory with the recruitment of cellular immunity and the induction of apoptosis, its scheme might suggest the possibility that NOP is able to produce immunomodulation. However, to reach significant levels of modulation, more sessions will be needed, as has recently been shown for 30% and 100% FiO_2_ after 5 weeks of regular sessions three times per week [11]. Isoprostane changes are parallel for both exposures, and lipid damage follows ROS production in both percentages (30% and 100%) of administered O_2_. 

Other recent data on platelet (CD41a)-, astrocyte (TMEM119)-, and neutrophil (CD66+)-related microparticle release after oxygen exposure (30% and 100%) [15] are consistent with this analysis.

The more potent initial inflammatory response triggered by exposure at 100% is also confirmed based on uric acid measurements. Circulating uric acid is a major aqueous antioxidant in humans, especially for peroxynitrites (ONOO–) in the hydrophilic environment. However, uric acid becomes a powerful pro-oxidant under hydrophobic conditions. Uric acid can induce intracellular and mitochondrial oxidative stress and stimulate expression of inflammatory cytokines [30]. 

Finally, although not significant, the creatinine pattern is consistent with other results of this study, especially ROS and uric acid. Indeed, creatine kinase is inhibited by ROS, especially H_2_O_2_ [31], while uric acid acts as an iron chelator in extracellular fluids, which is a co-factor to creatine kinase activity [30]. Evidently, urates may be related to ROS generation (particularly under oxidative stress conditions), for example, when interacting with peroxynitrite, resulting in the formation of active but less reactive products [50]. 

It can thus be assumed that the observed increase in the ROS level in plasma after moderate hyperoxia (FiO_2_ of 30%) is associated not with the elevation of oxidative stress but with the protective mechanism harnessed by the body during the neutralization of highly reactive peroxynitrite with uric acid [51]. The results obtained by Gu et al. also suggest that hyperoxia-induced peroxynitrite formation causes endothelial cell apoptosis, disrupting key survival pathways, and that blocking peroxynitrite formation prevents apoptosis.

### Limitations

Strengths:

− This study is to our knowledge one of the first to tackle the kinetics of responses to a single normobaric oxygen exposure at 30% and 100% of FiO_2_.
− The measurements were conducted until 48 h post exposure and putatively open the door to new possible therapeutic outcomes for oxygen administration protocols.
− The sampling was a standard plasmatic withdrawal, and the analysis is possible in a usual clinical setting without very specialized machinery or procedures.

Weaknesses:

− The number of subjects is limited, but the sample can be considered to be homogenous since all were healthy young students and gender balanced.
− The analysis was not made in the nucleus of the cells but in the plasma; this could be considered a weakness for some, but analysis in the nucleus would need a thoroughly different experimental setting.

## 4. Materials and Methods

### 4.1. Experimental Protocol

Fourteen healthy subjects (7 females and 7 males) volunteered for this study, after approval from the Bio-Ethical Committee for Research and Higher Education, Brussels (No. B200-2020-088), and written informed consent was obtained. All experimental procedures were conducted in accordance with the Declaration of Helsinki [52]. 

**Figure 7:**
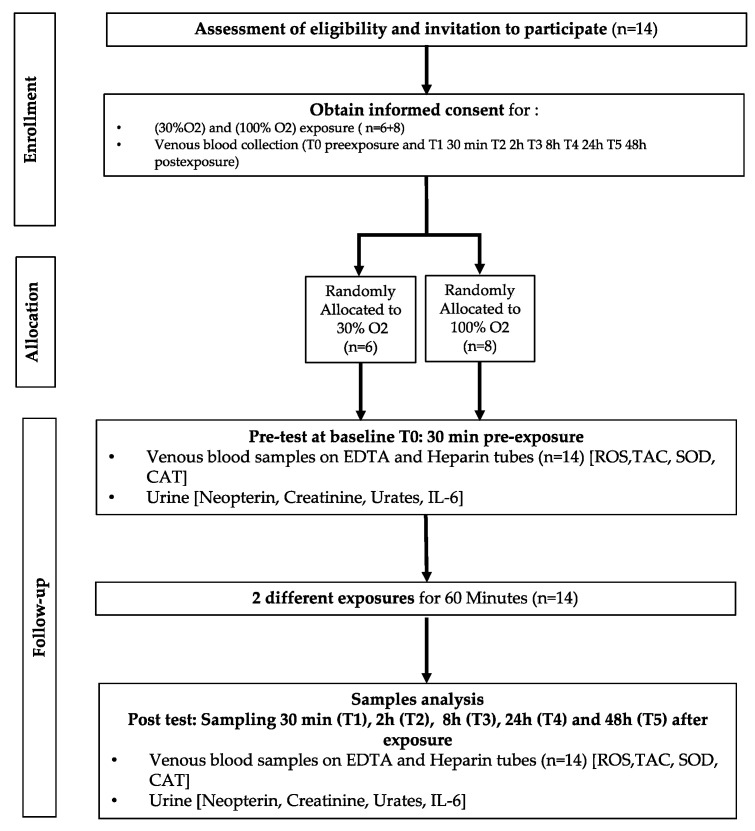
Experimental flowchart.

After medical screening to exclude any comorbidity, participants were prospectively randomized into 2 groups to receive oxygen at different FiO_2_ (Figure 7). As far as age (29.9 ± 11.1 years old (mean ± SD)), height (168.2 ± 9.37 cm) weight (64.4 ± 12.3 kg) BMI [22.7 ± 4.1 kg/m^2^], gender ratio, and health status are concerned, groups were comparable.

Oxygen was administered for 1 h by means of an orofacial nonrebreather mask with a reservoir with care being taken to fit and tighten the mask on the subject’s face. The mild hyperoxia group received 30% of oxygen (oxygen partial pressure (PO_2_): 0.3 bar; 300 hPa, n = 6) from a pressurized gas tank with a gas flow set at 10 L/min, while the high hyperoxia group received 100% of oxygen (PO_2_: 1.0 bar, 1000 hPa, n = 8) from an oxygen concentrator (NewLife Intensity, CAIRE Inc., Ball Ground, GA, USA).

Blood and urine samples were obtained before exposure (T0) and 30 min, 2 h, 8 h, 24 h, and 48 h after the end of oxygen administration.

Each blood sample consisted of approximately 15 mL of venous human blood collected in lithium heparin and EDTA tubes (Vacutainer, BD Diagnostic, Becton Dickinson, Italia S.p.a.). Plasma and red blood cells (RBCs) were separated by centrifugation (Eppendorf Centrifuge 5702R, Darmstadt Germany) at 1000× *g* at 4 °C for 10 min. The samples were then stored in multiple aliquots at −80 °C until assayed; analysis was performed within one month from collection.

Urine was collected by voluntary voiding in a sterile container and stored in multiple aliquots at −20 °C until assayed and thawed only before analysis.

### 4.2. Blood Sample Analysis

#### 4.2.1. Determination of ROS and TAC by Electron Paramagnetic Resonance (EPR)

An electron paramagnetic resonance instrument (E-Scan—Bruker BioSpin, GmbH, Rheinstetten, Germany) X-band, with a controller temperature at 37 °C interfaced to the spectrometer, was adopted for ROS production rate and total antioxidant capacity (TAC) as already performed by some of the authors [53,54,55,56]. The EPR measurements are highly reproducible, as previously demonstrated [57].

Briefly, for ROS detection, 50 µL of plasma were treated with an equal volume of CMH (1-hydroxy-3-methoxycarbonyl-2,2,5,5-tetramethylpyrrolidine); then 50 µL of this solution was placed inside a glass EPR capillary tube in the spectrometer cavity for data acquisition. A stable radical CP (3-carboxy-2,2,5,5-tetramethyl-1-pyrrolidinyloxy) was used as an external reference to convert ROS determinations in absolute quantitative values (μmol/min). For TAC, we used the spin trap DPPH· (2,2-diphenyl-1-picrylhydrazyl), a free radical compound soluble and stable in ethanol. All operations were performed in the dark to avoid photochemical effects on DPPH·, and a calibration curve was computed from pure Trolox-containing reactions. Finally, an equation was used to calculate the anti-oxidant capacity expressed in terms of Trolox equivalent antioxidant capacity (TAC, mM).

All EPR spectra were collected by adopting the same protocol and obtained by using software standardly supplied by Bruker (Billerica, MA, USA) (version 2.11, WinEPR System).

#### 4.2.2. Super-Oxide Dismutase (SOD), Catalase (CAT)

SOD, CAT plasmatic levels were measured by enzyme-linked immunosorbent assay (ELISA kits) according to the manufacturer’s instructions.

SOD activity was assessed by Cayman’s SOD assay kit (706002) that utilizes a tetrazolium salt for detection of superoxide radicals generated by xanthine oxidase and hypoxathine. 

One unit of SOD is defined as the amount of enzyme needed to exhibit 50% dismutation of the superoxide radical measured in change in absorbance (450 nm) per minute at 25° and pH 8.0.

CAT activity was assessed by Cayman’s assay kit (707002) that utilizes the peroxidic function of CAT. The method is based on the reaction of enzyme with methanol in presence of an optimal concentration of H_2_O_2_. 

The formaldehyde produced is measured colorimetrically with Purpald (540 nm) as chromogen. One unit of CAT is defined as the amount of enzyme that will cause the formation of 1 nmol of formaldehyde per minute at 25 °C.

All samples and standards were read by a microplate reader spectrophotometer (Infinite M200, Tecan Group Ltd., Männedorf, Switzerland). 

The determinations were assessed in duplicate, and the inter-assay coefficient of variation was in the range indicated by the manufacturer.

### 4.3. Urine Sample Analysis

#### 4.3.1. Lipid Peroxidation (8-iso-PGF2α)

Lipid peroxidation was assessed in urine by competitive immunoassay (Cayman Chemical, Ann Arbor, MI, USA) measuring 8-isoprostane (8-iso-PGF2α) concentration following the manufacturer’s recommendations. Briefly, 50 μL of urine was placed in a well plate with mouse monoclonal antibody. 

Therefore, 50 μL of 8-iso PGF2α-tracer and 8-iso PGF2α-antiserum were added and incubated for 18 h at 4 °C. After washing with buffer, 200 μL of Ellman’s reagent containing the substrate of acetylcholinesterase was added. 

The 8-iso-PGF2α concentrations were determined using a standard curve. Samples and standards were spectrophotometrically read at wavelengths between 405 and 420 nm.

#### 4.3.2. Interleukin-6

IL-6 levels were determined using the ELISA assay kit (ThermoFisher Scientific, Waltham, MA, USA), based on the double-antibody “sandwich” technique in accordance with the manufacturer’s instruction. All the above samples and standards were read by a microplate reader spectrophotometer (Infinite M200, Tecan Group Ltd., Männedorf, Switzerland). The determinations were assessed in duplicate, and the inter-assay coefficient of variation was in the range indicated by the manufacturer. 

#### 4.3.3. Creatinine, Neopterin, and Uric acid Concentrations

Urinary creatinine, neopterin, and uric acid concentrations were measured by the high-pressure liquid chromatography (HPLC) method, as previously described [58], by a Varian instrument (pump 240, autosampler ProStar 410) coupled to a UV–VIS detector (Shimadzu SPD 10-AV, λ = 240 nm for creatinine and uric acid; and JASCO FP-1520, λ_ex_ = 355 nm and at λ_em_ = 450 nm) for neopterin). 

After urine centrifugation at 13,000 rpm at 4 °C for 5 min, analytic separations were performed at 50 °C on a 5 µm Discovery C18 analytical column (250 × 4.6 mm I.D., Supelco, Sigma-Aldrich) at a flow rate of 0.9 mL/min. The calibration curves were linear over the range of 0.125–1 µmol/L, 0.625–20 mmol/L, and 1.25–10 mmol/L for neopterin, uric acid, and creatinine levels, respectively. The inter-assay and intra-assay coefficients of variation were <5%.

### 4.4. Statistical Analysis

The normality of the data was verified by means of the Shapiro–Wilk test, which allowed us to assume a Gaussian distribution. Compared to baseline (T0—pre-exposure), data were analyzed with a one-way ANOVA for repeated measures with Dunnett’s post hoc test for intragroup comparison. An unpaired *t* test was used for intergroup comparison. Taking the baseline measures as 100% (T0), percentage or fold changes were calculated for each oxygen protocol, allowing an appreciation of the magnitude of change rather than the absolute values. All data were presented as mean ± standard deviation (SD).

All statistical tests were performed using a standard computer statistical package, GraphPad Prism version 9.00 for Mac (GraphPad Software, San Diego, CA, USA). A threshold of *p* < 0.05 was considered statistically significant. 

## 5. Conclusions

This study suggests a biphasic response over time characterized by an initial “permissive oxidation” followed by the overexpression of inflammation. The antioxidant protection system seems not to be the leading actor in the first place. It corroborates the concept that cellular hormesis is dependent on complex multimodal processes driven by an increase in ROS, inflammation, metabolism, and probably gene expression. This last point needs to be better studied, especially the kinetics of enzymatic reactions related to the expression or repression of intracellular molecular biogenesis, since it is for us important to have sufficient understanding of the exposure/repetition-related leading mechanisms in order to optimize the outcome. This would allow for the definition of proper training and therapeutic or rehabilitation protocols aiming at a more targeted use of oxygen.

## Data Availability

Data are available upon request from the authors.

## Abbreviations

FIH	Factor inhibiting HIF
PHD	Prolyl hydroxylase domain
REST	Repressor Element 1-Silencing Transcription factor
CAT	Catalase
FiO2	Inspired fraction of oxygen
HIF-1α	Hypoxia Inducible Factor-1α
IL-6	Interleukin-6
NOP	Normobaric oxygen paradox
NF-κB	Nuclear factor-kappa B
NRF2	Nuclear Factor Erythroid 2 Related–Factor 2
PO2	Oxygen partial pressure
ROS	Reactive oxygen species
SOD	Superoxide dismutase
TAC	Total antioxidant capacity
HRE	Hypoxia responsive element

## References

[1] Poulton S.W., Bekker A., Cumming V.M., Zerkle A.L., Canfield D.E., Johnston D.T. (2021). A 200-million-year delay in permanent atmospheric oxygenation. Nature.

[2] Bitterman H. (2009). Bench-to-bedside review: Oxygen as a drug. Crit. Care.

[3] Nakane M. (2020). Biological effects of the oxygen molecule in critically ill patients. J. Intensive Care.

[4] van Vliet T., Demaria F.C.M. (2021). To breathe or not to breathe: Understanding how oxygen sensing contributes to age-related phenotypes. Ageing Res. Rev..

[5] Fu Q., Duan R., Sun Y., Li Q. (2022). Hyperbaric oxygen therapy for healthy aging: From mechanisms to therapeutics. Redox Biol..

[6] Okada K., Mori D., Makii Y., Nakamoto H., Murahashi Y., Yano F., Chang S.H., Taniguchi Y., Kobayashi H., Semba H. (2020). Hypoxia-inducible factor-1 alpha maintains mouse articular cartilage through suppression of NF-κB signaling. Sci. Rep..

[7] Serebrovska Z.O., Serebrovska T.V., Kholin V.A., Tumanovska L.V., Shysh A.M., Pashevin D.A., Goncharov S.V., Stroy D., Grib O.N., Shatylo V.B. (2019). Intermittent Hypoxia-Hyperoxia Training Improves Cognitive Function and Decreases Circulating Biomarkers of Alzheimer’s Disease in Patients with Mild Cognitive Impairment: A Pilot Study. Int. J. Mol. Sci..

[8] Burtscher J., Mallet R.T., Burtscher M., Millet G.P. (2021). Hypoxia and brain aging: Neurodegeneration or neuroprotection?. Ageing Res. Rev..

[9] Bestavashvili A., Glazachev O., Bestavashvili A., Suvorov A., Zhang Y., Zhang X., Rozhkov A., Kuznetsova N., Pavlov C., Glushenkov D. (2022). Intermittent Hypoxic-Hyperoxic Exposures Effects in Patients with Metabolic Syndrome: Correction of Cardiovascular and Metabolic Profile. Biomedicines.

[10] Matta A., Nader V., Lebrin M., Gross F., Prats A.C., Cussac D., Galinier M., Roncalli J. (2022). Pre-Conditioning Methods and Novel Approaches with Mesenchymal Stem Cells Therapy in Cardiovascular Disease. Cells.

[11] Balestra C., Lambrechts K., Mrakic-Sposta S., Vezzoli A., Levenez M., Germonpre P., Virgili F., Bosco G., Lafere P. (2021). Hypoxic and Hyperoxic Breathing as a Complement to Low-Intensity Physical Exercise Programs: A Proof-of-Principle Study. Int. J. Mol. Sci..

[12] Balestra C., Theunissen S., Papadopoulou V., Le Mener C., Germonpre P., Guerrero F., Lafere P. (2016). Pre-dive Whole-Body Vibration Better Reduces Decompression-Induced Vascular Gas Emboli than Oxygenation or a Combination of Both. Front. Physiol..

[13] Webb J.T., Pilmanis A.A. (2011). Fifty years of decompression sickness research at Brooks AFB, TX: 1960-2010. Aviat. Space Env. Med..

[14] Sannigrahi P., Sushree S.K., Agarwal A. (2018). Aeromedical Concerns and Lessons Learnt during Oxygen Jump at Dolma Sampa. Indian J. Aerosp. Med..

[15] Balestra C., Arya A.K., Leveque C., Virgili F., Germonpre P., Lambrechts K., Lafere P., Thom S.R. (2022). Varying Oxygen Partial Pressure Elicits Blood-Borne Microparticles Expressing Different Cell-Specific Proteins-Toward a Targeted Use of Oxygen?. Int. J. Mol. Sci..

[16] Halliwell B., Gutteridge J.M.C. (2015). Free Radicals in Biology and Medicine.

[17] Sies H., Belousov V.V., Chandel N.S., Davies M.J., Jones D.P., Mann G.E., Murphy M.P., Yamamoto M., Winterbourn C. (2022). Defining roles of specific reactive oxygen species (ROS) in cell biology and physiology. Nat. Rev. Mol. Cell Biol..

[18] La Sala L., Tagliabue E., Mrakic-Sposta S., Uccellatore A.C., Senesi P., Terruzzi I., Trabucchi E., Rossi-Bernardi L., Luzi L. (2022). Lower miR-21/ROS/HNE levels associate with lower glycemia after habit-intervention: DIAPASON study 1-year later. Cardiovasc. Diabetol..

[19] Cova E., Pandolfi L., Colombo M., Frangipane V., Inghilleri S., Morosini M., Mrakic-Sposta S., Moretti S., Monti M., Pignochino Y. (2019). Pemetrexed-loaded nanoparticles targeted to malignant pleural mesothelioma cells: An in vitro study. Int. J. Nanomed..

[20] Mrakic-Sposta S., Vezzoli A., Maderna L., Gregorini F., Montorsi M., Moretti S., Greco F., Cova E., Gussoni M. (2018). R(+)-Thioctic Acid Effects on Oxidative Stress and Peripheral Neuropathy in Type II Diabetic Patients: Preliminary Results by Electron Paramagnetic Resonance and Electroneurography. Oxid. Med. Cell Longev..

[21] Calabrese E.J., Mattson M.P. (2017). How does hormesis impact biology, toxicology, and medicine?. NPJ Aging Mech. Dis..

[22] Lennicke C., Cochemé H.M. (2021). Redox metabolism: ROS as specific molecular regulators of cell signaling and function. Mol. Cell.

[23] Chelombitko M.A. (2018). Role of Reactive Oxygen Species in Inflammation: A Minireview. Mosc. Univ. Biol. Sci. Bull..

[24] Hadanny A., Efrati S. (2020). The Hyperoxic-Hypoxic Paradox. Biomolecules.

[25] Balestra C., Germonpré P., Poortmans J.R., Marroni A. (2006). Serum erythropoietin levels in healthy humans after a short period of normobaric and hyperbaric oxygen breathing: The "normobaric oxygen paradox". J. Appl. Physiol..

[26] Cimino F., Balestra C., Germonpre P., De Bels D., Tillmans F., Saija A., Speciale A., Virgili F. (2012). Pulsed high oxygen induces a hypoxic-like response in human umbilical endothelial cells and in humans. J. Appl. Physiol..

[27] Khalife M., Ben Aziz M., Balestra C., Valsamis J., Sosnowski M. (2021). Physiological and Clinical Impact of Repeated Inhaled Oxygen Variation on Erythropoietin Levels in Patients After Surgery. Front. Physiol..

[28] Lafere P., Schubert T., De Bels D., Germonpre P., Balestra C. (2013). Can the normobaric oxygen paradox (NOP) increase reticulocyte count after traumatic hip surgery?. J. Clin. Anesth..

[29] Fratantonio D., Virgili F., Zucchi A., Lambrechts K., Latronico T., Lafere P., Germonpre P., Balestra C. (2021). Increasing Oxygen Partial Pressures Induce a Distinct Transcriptional Response in Human PBMC: A Pilot Study on the "Normobaric Oxygen Paradox". Int. J. Mol. Sci..

[30] Calabrese E. (2018). Hormesis: Path and Progression to Significance. Int. J. Mol. Sci..

[31] Franceschi C., Bonafè M., Valensin S., Olivieri F., De Luca M., Ottaviani E., De Benedictis G. (2000). Inflamm-aging. An evolutionary perspective on immunosenescence. Ann. N. Y. Acad. Sci..

[32] Franceschi C., Campisi J. (2014). Chronic inflammation (inflammaging) and its potential contribution to age-associated diseases. J. Gerontol. A Biol. Sci. Med. Sci..

[33] Furman D., Campisi J., Verdin E., Carrera-Bastos P., Targ S., Franceschi C., Ferrucci L., Gilroy D.W., Fasano A., Miller G.W. (2019). Chronic inflammation in the etiology of disease across the life span. Nat. Med..

[34] Akhigbe R., Ajayi A. (2021). The impact of reactive oxygen species in the development of cardiometabolic disorders: A review. Lipids Health Dis..

[35] Checa J., Aran J.M. (2020). Reactive Oxygen Species: Drivers of Physiological and Pathological Processes. J. Inflamm. Res..

[36] Balestra C., Kot J. (2021). Oxygen: A Stimulus, Not "Only" a Drug. Medicina.

[37] Rocco M., D’Itri L., De Bels D., Corazza F., Balestra C. (2014). The "normobaric oxygen paradox": A new tool for the anesthetist?. Minerva Anestesiol..

[38] Bel’skaya L.V., Kosenok V.K., Sarf E.A. (2017). Chronophysiological features of the normal mineral composition of human saliva. Arch. Oral. Biol..

[39] Teng R.J., Jing X., Martin D.P., Hogg N., Haefke A., Konduri G.G., Day B.W., Naylor S., Pritchard K.A. (2021). N-acetyl-lysyltyrosylcysteine amide, a novel systems pharmacology agent, reduces bronchopulmonary dysplasia in hyperoxic neonatal rat pups. Free Radic. Biol. Med..

[40] Chen C., He M., Li X., Yu L., Liu Y., Yang Y., Li L., Jia J., Li B. (2022). H2O2/DEM-Promoted Maft Promoter Demethylation Drives Nrf2/ARE Activation in Zebrafish. Life.

[41] Semenza G.L. (2000). HIF-1: Mediator of physiological and pathophysiological responses to hypoxia. J. Appl. Physiol..

[42] Cavadas M.A.S., Mesnieres M., Crifo B., Manresa M.C., Selfridge A.C., Scholz C.C., Cummins E.P., Cheong A., Taylor C.T. (2015). REST mediates resolution of HIF-dependent gene expression in prolonged hypoxia. Sci. Rep..

[43] Cavadas M.A.S., Mesnieres M., Crifo B., Manresa M.C., Selfridge A.C., Keogh C.E., Fabian Z., Scholz C.C., Nolan K.A., Rocha L.M.A. (2016). REST is a hypoxia-responsive transcriptional repressor. Sci. Rep..

[44] De Bels D., Corazza F., Germonpre P., Balestra C. (2010). The normobaric oxygen paradox: A novel way to administer oxygen as an adjuvant treatment for cancer?. Med. Hypotheses.

[45] Sies H. (2014). Role of Metabolic H2O2 Generation. J. Biol. Chem..

[46] Basang Z., Zhang S., Yang L., Quzong D., Li Y., Ma Y., Hao M., Pu W., Liu X., Xie H. (2021). Correlation of DNA methylation patterns to the phenotypic features of Tibetan elite alpinists in extreme hypoxia. J. Genet. Genom..

[47] Wilson J.W., Shakir D., Batie M., Frost M., Rocha S. (2020). Oxygen-sensing mechanisms in cells. FEBS J..

[48] Kobayashi E.H., Suzuki T., Funayama R., Nagashima T., Hayashi M., Sekine H., Tanaka N., Moriguchi T., Motohashi H., Nakayama K. (2016). Nrf2 suppresses macrophage inflammatory response by blocking proinflammatory cytokine transcription. Nat. Commun..

[49] Gao W., Guo L., Yang Y., Wang Y., Xia S., Gong H., Zhang B.K., Yan M. (2021). Dissecting the Crosstalk Between Nrf2 and NF-κB Response Pathways in Drug-Induced Toxicity. Front. Cell Dev. Biol..

[50] Kuzkaya N., Weissmann N., Harrison D.G., Dikalov S. (2005). Interactions of peroxynitrite with uric acid in the presence of ascorbate and thiols: Implications for uncoupling endothelial nitric oxide synthase. Biochem. Pharm..

[51] Gu X., El-Remessy A.B., Brooks S.E., Al-Shabrawey M., Tsai N.T., Caldwell R.B. (2003). Hyperoxia induces retinal vascular endothelial cell apoptosis through formation of peroxynitrite. Am. J. Physiol. Cell Physiol..

[52] World Medical A. (2013). World Medical Association Declaration of Helsinki: Ethical principles for medical research involving human subjects. JAMA.

[53] Mrakic-Sposta S., Vezzoli A., D’Alessandro F., Paganini M., Dellanoce C., Cialoni D., Bosco G. (2020). Change in Oxidative Stress Biomarkers During 30 Days in Saturation Dive: A Pilot Study. Int. J. Env. Res. Public Health.

[54] Moretti S., Mrakic-Sposta S., Roncoroni L., Vezzoli A., Dellanoce C., Monguzzi E., Branchi F., Ferretti F., Lombardo V., Doneda L. (2018). Oxidative stress as a biomarker for monitoring treated celiac disease. Clin. Transl. Gastroenterol..

[55] Mrakic-Sposta S., Vezzoli A., Rizzato A., Della Noce C., Malacrida S., Montorsi M., Paganini M., Cancellara P., Bosco G. (2019). Oxidative stress assessment in breath-hold diving. Eur. J. Appl. Physiol..

[56] Bosco G., Rizzato A., Quartesan S., Camporesi E., Mrakic-Sposta S., Moretti S., Balestra C., Rubini A. (2018). Spirometry and oxidative stress after rebreather diving in warm water. Undersea Hyperb. Med..

[57] Mrakic-Sposta S., Gussoni M., Montorsi M., Porcelli S., Vezzoli A. (2014). A quantitative method to monitor reactive oxygen species production by electron paramagnetic resonance in physiological and pathological conditions. Oxid. Med. Cell Longev..

[58] Bosco G., Paganini M., Giacon T.A., Oppio A., Vezzoli A., Dellanoce C., Moro T., Paoli A., Zanotti F., Zavan B. (2021). Oxidative Stress and Inflammation, MicroRNA, and Hemoglobin Variations after Administration of Oxygen at Different Pressures and Concentrations: A Randomized Trial. Int. J. Env. Res. Public Health.

